# Reevaluation for clinical manifestations of HTLV-I-seropositive patients with Sjögren’s syndrome

**DOI:** 10.1186/s12891-015-0773-1

**Published:** 2015-11-04

**Authors:** Hideki Nakamura, Toshimasa Shimizu, Yukinori Takagi, Yoshiko Takahashi, Yoshiro Horai, Yoshikazu Nakashima, Shuntaro Sato, Hirokazu Shiraishi, Tatsufumi Nakamura, Junya Fukuoka, Takashi Nakamura, Atsushi Kawakami

**Affiliations:** Unit of Translational Medicine, Department of Immunology and Rheumatology, Nagasaki University Graduate School of Biomedical Sciences, 1-7-1 Sakamoto, Nagasaki City, Nagasaki 852-8501 Japan; Department of Radiology and Cancer Biology, Nagasaki University School of Dentistry, Nagasaki, Japan; Clinical Research Center, Nagasaki University Hospital, Nagasaki, Japan; Unit of Translational Medicine, Department of Clinical Neuroscience and Neurology, Nagasaki University Graduate School of Biomedical Sciences, Nagasaki, Japan; Department of Human Community, Faculty of Social Welfare, Nagasaki International University, Nagasaki, Japan; Department of Pathology, Nagasaki University Hospital, Nagasaki, Japan

**Keywords:** Sjögren’s syndrome, HTLV-I, AECG criteria, Minor salivary gland biopsy

## Abstract

**Background:**

The aim of the study was to reassess the prevalence and characteristics of human T lymphotropic virus type I (HTLV-I)-associated Sjögren’s syndrome (SS) and SS in HTLV-I-associated myelopathy (HAM) based on the American European Consensus Group (AECG) criteria in HTLV-I endemic area, Nagasaki prefecture.

**Methods:**

The 349 patients who underwent a minor salivary gland biopsy (MSGB) for suspected SS were retrospectively classified by AECG classification criteria and divided with or without anti-HTLV-I antibody.

**Results:**

The HTLV-I data-available 294 patients were investigated. One hundred-seventy patients were classified as SS and 26.5 % were HTLV-I-seropositive. We have included 26 patients with HTLV-I-associated myelopathy (HAM) and 38.5 % were classified as having SS. The prevalences of ANA and anti-SS-A/Ro antibody of HAM + SS were significantly low compared to the HTLV-I asymptomatic carriers (AC) with SS and the HTLV-I-seronegative SS patients, although lacrimal dysfunction tended to be high in HAM + SS and significantly high in AC + SS patients compared with the patients with HTLV-I-seronegative SS. The focus scores of MSGB in the HAM + SS patients were similar to those of the AC + SS patients and the HTLV-I-seronegative patients with SS. Among the MSGB-positive patients, there was a low prevalence of ANA in the HAM + SS patients. Similar results were obtained in case of anti-SS-A/Ro or SS-B/La antibody.

**Conclusion:**

In HTLV-I endemic area, high prevalence of anti-HTLV-I antibody among SS as well as the characteristics of HAM + SS and AC + SS was still determined by AECG classification criteria.

## Background

Human T lymphotropic virus type I (HTLV-I), one of the human retroviruses, is known to be a causative agent of HTLV-I-associated myelopathy (HAM)/tropical spastic paraparesis (TSP) and adult-T cell leukemia (ATL) [1, 2]. Sjögren’s syndrome (SS) is an autoimmune disorder characterized by sicca symptoms due to mononuclear cell (MNC) infiltration into the salivary glands or lacrimal glands [3–5]. The association between HTLV-I and SS was discussed in the 1990s, and epidemiological evidence of the association has been published [6–9]. We also reported a high prevalence of SS among individuals with HAM [10, 11], which strongly supports the idea that HTLV-I is involved in the pathogenesis of SS in a subset of SS patients in HTLV-I endemic areas.

However, we should note that the diagnosis of SS in the 1990s was performed using the Preliminary European classification criteria [12] published in 1993 (the preliminary classification criteria). These criteria consist of six items, and the diagnosis of primary SS (pSS) is determined by the presence of four of the six items. The classification criteria released in 2002 by the American European Consensus Group (AECG; i.e., the AECG classification criteria) [13] are now used to classify pSS and secondary SS (sSS). The AECG classification criteria [12] have revised rules to classify primary SS: namely, positive histopathology or serology is necessary when all six items are factors for classification. However, the presence of three items among four objective items also allows the classification of a patient as having primary SS. In addition, the newly proposed American College of Rheumatology (ACR) classification criteria [14] provide a simpler diagnostic method because a minimum of two out of three items is sufficient for the diagnosis of SS.

In our previous studies [10, 11], the exocrine dysfunction of the patients with SS was similar regardless of HTLV-I infection. However, the prevalence of anti-nuclear antibody (ANA) and anti-SS-A/Ro, SS-B/La antibodies of HAM patients with SS was lower than that of asymptomatic HTLV-I carriers (ACs) with SS and HTLV-I-seronegative SS when the preliminary criteria were used. We therefore reevaluated the suspected SS patients by using the AECG classification criteria. Although this historical cohort study was conducted retrospectively with cases from a single center in an HTLV-I endemic area, i.e., Nagasaki prefecture in Japan, our findings can provide a current perspective with regard to the clinical characteristics of HTLV-I-seropositive patients with SS.

## Methods

### Patients

We enrolled 349 patients who underwent a minor salivary gland biopsy (MSGB) for suspected SS between 1995 and 2015 at Nagasaki University Hospital. The availability of data regarding ocular signs including Rose-Bengal staining and fluorescein testing results was too low to use the ACR classification criteria [13] released in 2012, and therefore we used the AECG classification criteria [12]. The classifications of pSS and sSS were accomplished in accord with the revised rule in the AECG criteria, respectively.

**Fig. 1:**
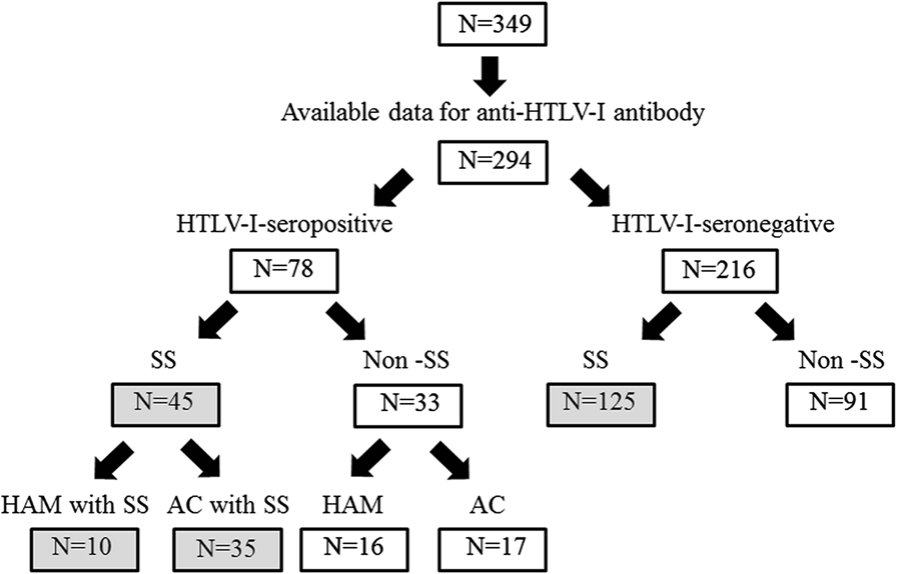
Patients with suspected SS who underwent a minor salivary gland biopsy (MSGB). All patients who underwent an MSGB were divided into subgroups according to the revised classification criteria determined by the American European Consensus Group (AECG). SS: Sjögren’s syndrome, AC: HTLV-I asymptomatic carrier. Shaded squares indicate the patients with SS

In accord with the AECG exclusion criteria, we excluded all cases that involved radiation treatment, evident hepatitis induced by hepatitis type C, acquired immunodeficiency disease, lymphoma, sarcoidosis, graft versus host disease, or the use of anticholinergic drugs. Among the 349 patients, 294 patients had available data for anti-HTLV-I antibody (Fig. 1). Among these 294 patients, the cases of 45 patients were complicated with other connective tissue diseases including 21 rheumatoid arthritis, 6 lupus erythematosus, 4 systemic sclerosis, 4 Graves’ disease, 2 multiple sclerosis, 2 mixed connective tissue disease, 1 polymyositis, 1 aortitis syndrome and 1 polyarteritis nodosa.

The study was conducted in accordance with the human experimental guidelines of our institution and was approved by the Institutional Review Board of Nagasaki University. Regarding the handling of patient data that were obtained before the acquisition of written informed consent, we present a brief overview of our study on a Nagasaki University Hospital website in accord with clinical study guidelines issued by our hospital. These records of patients before acquisition of written informed consent were anonymized prior to analysis. The division of enrolled patients according to the AECG classification criteria is summarized in Fig. 1. The diagnosis of HAM was determined by the criteria published by Osame et al. [15].

### Serological tests and exocrine dysfunction

Detailed explanations of the serological tests and exocrine dysfunction evaluation are described in our previous papers [10, 14]. Briefly, antibodies to SS-A/Ro and SS-B/La antigens were each measured by an enzyme-linked immunosorbent assay (ELISA) (Mesacup SS-A/Ro test, normal range: 10–30 and Mesacup SS-B/La test, normal range: 15–25; MBL, Nagoya, Japan). With respect to screening for anti-HTLV-I antibodies, we performed an ELISA or particle agglutination assay (Serodia-ATL kit, Eisai, Tokyo). As of 1996, a chemiluminescent enzyme immunoassay (normal range: 1.0 cutoff index; Fujirebio, Tokyo) was used.

For the confirmation of HTLV-I infection among the HTLV-I-seropositive patients, an immunoblotting kit (Problot-HTLV-I, Fujirebio) that included HTLV-I antigens such as three gag proteins and 1 env protein was used. To measure the patients’ tears and saliva secretion, Schirmer’s test and the Saxon test were performed. In the AECG classification criteria, unstimulated saliva is used. Instead of the unstimulated saliva volume, we used the Saxon test. Results from the sialography were assessed by using the classification determined by Rubin and Holt [16].

### Minor labial salivary gland biopsy and sialography

Each MSGB biopsy was performed to obtain labial salivary glands under a local anesthetic. The grading of MNC infiltration in the salivary glands was defined by Chisholm and Mason [17]. The appearance of at least one focus of MNCs in 4-mm^2^ sections was defined as grade 3. The focus score (FS) was determined as described by Greenspan et al. [18]. Sialography was performed by the insertion of a catheter into Stensen’s duct [19]. After that, a contrast fluid was slowly injected into the Stensen’s duct, followed by image detection.

### Statistical analysis

For the comparison of background information and clinical characteristics of the enrolled patients, Welch’s *t-*test or Fisher’s exact probability test was used. Fisher’s exact probability test was also used for the comparison of objective items in the AECG classification criteria according to MSGB grading. For the statistical analysis for the dot plot of FS values, Mann-Whitney’s *U*-test was used. *P*-values <0.05 were accepted as significant. Interquartile ranges (IQRs) were also calculated for the FS dot plot. These analyses were exploratory in nature, and no adjustment for multiple testing was applied.

## Results

### Prevalence of SS in HTLV-I-seropositive subjects according to the AECG classification criteria

We divided the 294 enrolled patients whose anti-HTLV-I antibody data were available into the 78 HTLV-I-seropositive and 216 HTLV-I-seronegative patients. The patients were also separated into SS and non-SS patients according to the AECG classification criteria. Forty-five HTLV-I-seropositive and 125 HTLV-I-seronegative patients were confirmed to have SS, and thus 26.5 % of the 170 patients with SS were HTLV-I-seropositive. In addition, among the 26 patients with HAM, 10 patients (38.5 %) were classified as having SS.

### Background elements according to the AECG classification criteria for HTLV-I-seropositive and -seronegative patients with SS

We divided the enrolled SS patients into three groups: HAM patients with SS, asymptomatic HTLV-I carriers (AC) with SS, and HTLV-I-seronegative patients with SS (Table 1). The prevalence of ANA as well as anti-SS-A/Ro or SS-B/La antibody among the HAM + SS patients was lower than those among the AC + SS patients or the HTLV-I-seronegative patients with SS. However, the prevalence of these antibodies was not significantly different between the AC + SS patients and the HTLV-I-seronegative patients with SS. Contrarily, the prevalence of ocular signs among the HAM + SS patients was significantly higher than that among the HTLV-I-seronegative patients with SS. A similar tendency was also found in the comparison with the AC + SS patients. The prevalence of the ANA centromere pattern was significantly different between the AC + SS patients and the HTLV-I-seronegative patients with SS.

**Table 1:** Clinical characteristics of Sjögren’s syndrome (SS) with HAM, whole HAM, SS with asymptomatic carrier and HTLV-I-seronegative SS

	HAM + SS (*n* = 10) (A)	AC + SS (*n* = 35) (B)	SS negative for anti-HTLV-I Ab (*n* = 125) (C)	*p*-value (A) vs. (B)	*p*-value (A) vs. (C)	*p*-value (B) vs. (C)
Age (yrs mean ± SD)	64.0 ± 9.8	61.8 ± 12.4	58.1 ± 13.0	0.279	0.049	0.064
Sex (M/F)	0/10	4/31	4/12	0.311	0.725	0.055
Dry eye (%)	8/10 (80.0)	23/33 (69.7)	79/120 (65.8)	0.421	0.296	0.423
Dry mouth (%)	7/10 (70.0)	29/33 (87.9)	101/123 (82.1)	0.96	0.908	0.309
MSGB grade 3 (%)	6/10 (60.0)	14/35 (40.0)	44/125 (35.2)	0.223	0.102	0.37
MSGB grade 4 (%)	4/10 (40.0)	19/35 (54.3)	69/125 (55.2)	0.331	0.274	0.615
Anti-nuclear antibody (%)	4/10 (40.0)	29/35 (82.9)	110/125 (88.0)	0.013	<0.001	0.859
Centromere pattern in positive ANA (%)	0/4 (0.0)	2/29 (6.9)	28/110 (25.4)	0.769	0.318	0.021
Anti-SS-A/Ro or SS-B/La antibody (%)	3/10 (30.0)	23/35 (65.7)	88/125 (70.4)	0.044	0.014	0.772
Rheumatoid factor (%)	3/5 (60.0)	12/24 (50.0)	48/90 (53.3)	0.535	0.57	0.952
Serum IgG (mg/dL; mean ± SD)	2349.2 ± 1015.5	2164.5 ± 1056.2	1922.3 ± 751.4	0.32	0.124	0.113
Schirmer’s test or Rose-Bengal test/Fluorescein (%)	9/10 (90.0)	28/31 (90.3)	69/115 (60.0)	0.754	0.055	<0.001
Saxon test or sialography (%)	8/10 (80.0)	26/30 (86.7)	101/116 (87.1)	0.847	0.866	0.656

Results from the minor labial salivary gland biopsy were determined as described by Chisholm & Mason [17]. The presence of at least one focus consisting of mononuclear cell aggregation was determined as grade 3. The *p*-values were calculated by Welch’s *t*-test or Fisher’s exact probability test. MSGB: minor salivary gland biopsy

### Differences in the focus scores of MSGB specimens among the HTLV-I-seropositive and -seronegative patients with SS

**Fig. 2:**
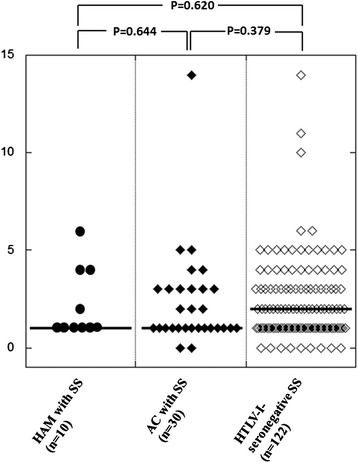
Distribution of focus scores (FS) among the three patient groups. The MSGB focus scores were determined by the method of Greenspan et al. [18]. The presence of at least one focus consisting of mononuclear cell (MNC) aggregation was determined as grade 3. Bars indicate the median value of each column. The significance of differences was calculated by Mann-Whitney’s *U*-test. NS; not significant AC: HTLV-I asymptomatic carrier

The degree of MNC infiltration determined by MSGB is illustrated in Fig. 2 by the plot of FS values of the three patient groups. The FS values of the HAM + SS patients (median [IQR] = 1.0 [1.0–3.5]) were not significantly different from those of the AC + SS patients (median [IQR] = 1.0 [1.0–3.0]) or the HTLV-I-seronegative patients with SS (median [IQR] = 2.0 [1.0–3.0]).

### The prevalence of the objective items in the MSGB-positive patients with SS

**Fig. 3:**
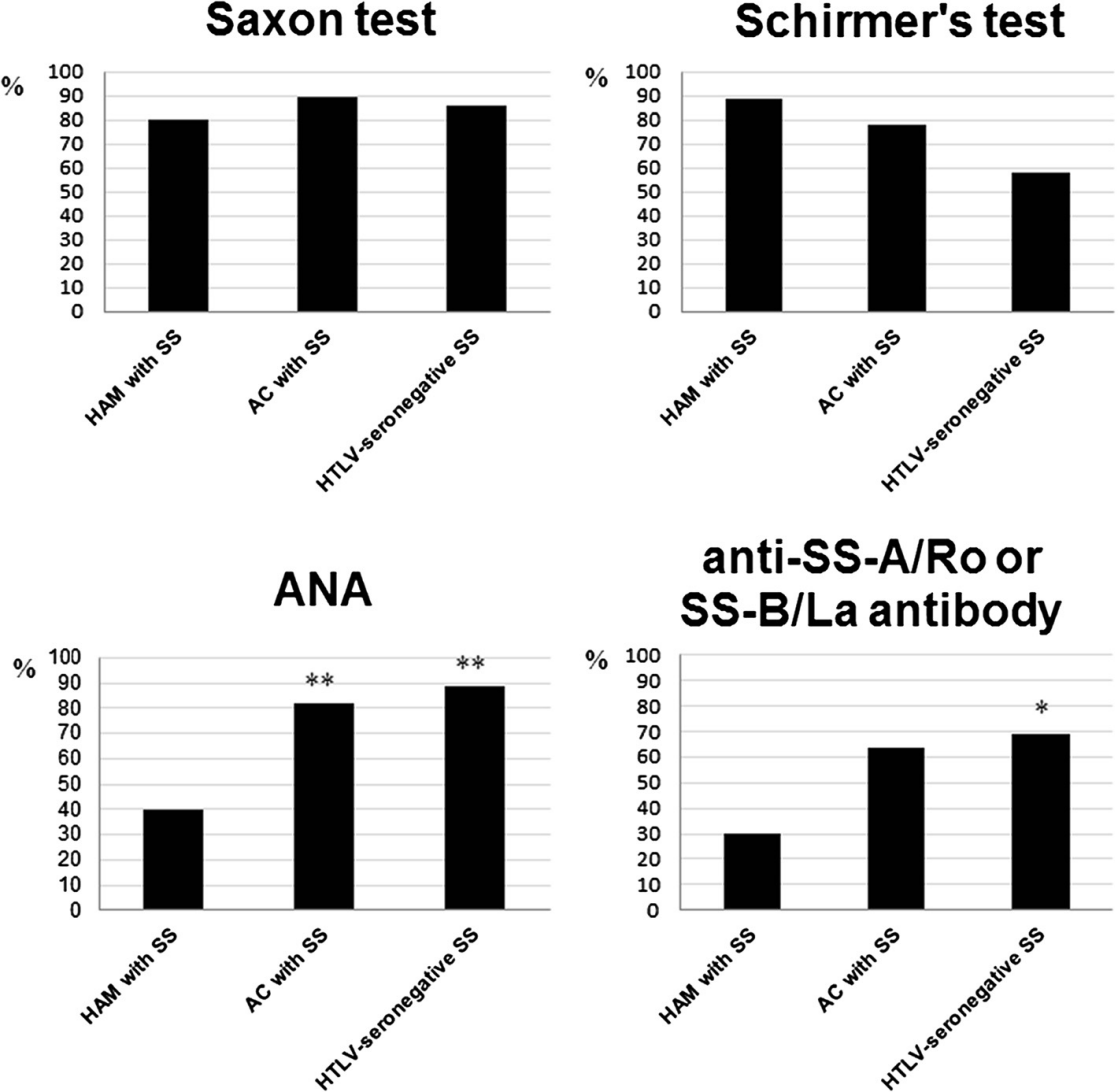
Prevalence of objective items in SS patients with grades 3 or 4 among the four patient groups. The prevalence of each item was determined by Chisholm & Mason grading [17]. The presence of at least one focus consisting of MNC aggregation was determined as grade 3. The positive rate of four objective items is shown. The significance of differences was assessed using Fisher’s exact probability test. (*p < 0.05 vs. HAM + SS, **p < 0.01 vs. HAM + SS) AC: HTLV-I asymptomatic carrier, ANA: anti-nuclear antibody

Lastly, we compared the prevalence of four objective items in the cases that were MSGB-positive in the AECG classification criteria among the three groups with patients with SS (Fig. 3). There was no significant difference among the Saxon test and Schirmer’s test results among the groups.

The prevalence of ANA among the HAM + SS patients was significantly lower than those of the AC + SS patients and the HTLV-seronegative patients with SS (*p* < 0.001). In addition, the prevalence of anti-SS-A/Ro or SS-B/La antibody among the HAM + SS patients was significantly lower than that of the HTLV-seronegative patients with SS (*p* = 0.013), and there was a tendency for this tendency to be lower compared with the AC + SS patients (*p* = 0.061) The prevalences were determined by Fisher’s exact probability test.

### Classification of SS in the patients with HAM according to the AECG criteria and the preliminary classification criteria

With regard to the 10 HAM patients who were classified as having SS, their profiles based on the AECG classification criteria are shown in Table 2. All 10 patients also fulfilled the preliminary classification criteria. With respect to the AECG classification criteria, seven patients fulfilled four items, two patients fulfilled five items, and one patient fulfilled six items. As shown in Table 1, the positive rate of anti-SS-A/Ro or SS-B/La antibody was 3 of these 10 patients (30.0 %).

**Table 2:**
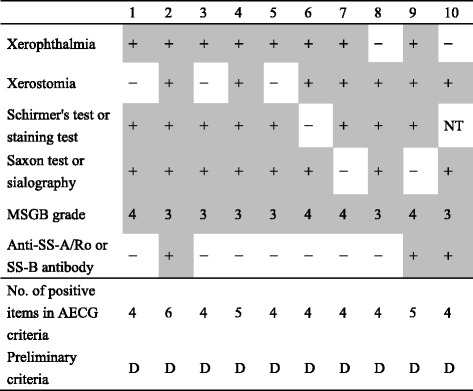
The 10 HAM patients with Sjögren’s syndrome (SS) according to the AECG classification criteria

The classification of SS was performed using both the AECG and preliminary classification criteria. With respect to the concordance number for the AECG classification criteria, positive numbers are shown. The shaded region represents the positive items according to the AECG classification criteria. D: Definitive diagnosis of SS that was determined in the preliminary criteria

## Discussion

After Green et al. [20] demonstrated in 1989 that HTLV-I *tax* transgenic mice showed SS-like sialadenitis, the association between HTLV-I and SS was studied. Epidemiological studies by Nagasaki [6, 21] showed distinct evidence with regard to the involvement of HTLV-I in SS as one of the environmental factors. In our studies of complications of SS in HAM patients [10, 14], we used the preliminary criteria [11] released in 1993. Although in the preliminary criteria four of the six items are required to classify SS, there was no limitation regarding which four items.

The clinical and pathological manifestations of the HTLV-I-seropositive patients with SS according to the AECG classification criteria are presented herein. In Fig. 1, the prevalence of HTLV-I among the confirmed SS patients was 45 of 170 (26.4 %). Since it is known that approx. 1 % of the entire population of Japan has anti-HTLV-I antibody [22, 23], the 26.5 % seropositivity in the present study’s SS patients is thought to be higher than that of the Japanese general population. In this regard, we should emphasize that the enrolled patients in this study are considerably different from the general population. We should also note that the enrolled subjects were suspected of having SS and that the classification of the enrolled subjects was based on salivary gland biopsies, indicating that this biased classification influences the interpretation of the present data.

The prevalence of ANA and anti-SS-A/Ro or SS-B/La antibodies determined by the AECG classification criteria was significantly low in the present population’s HAM + SS patients compared to the AC + SS patients and the HTLV-I-seronegative patients with SS, which is in accord with the results [10, 14] determined by the preliminary classification criteria. Contrarily, the prevalence of ocular signs including Schirmer’s test or staining tests in the HAM + SS patients was higher than that in the HTLV-I-seronegative patients with SS. These results suggest that HAM patients with SS have a high prevalence of ocular inflammation and a low prevalence of serological abnormalities.

With regard to the prevalence of SS among patients with HAM, our 2000 study revealed that the cases of 13 of 20 patients with HAM (65 %) were complicated with SS according to the preliminary classification criteria [14]. There is a tendency for a difference (*p* = 0.068 determined by Fisher’s exact probability test) between the 65 % we reported in 2000 and the 38.5 % we observed in the present 2015 study (i.e., among the 26 patients with HAM, 10 patients [38.5 %] were classified as having SS); however, this difference is not significant even though there is a 15-year difference in the dates of the two studies and differences in the classification approach.

The degree of cell infiltration into minor salivary glands (MSGs) determined by the FS established by Greenspan et al. [18] was equally high in the present HAM + SS patients compared to that in the AC + SS patients and the HTLV-I-seronegative patients with SS. As far as the positive MSGB cases were concerned, a low prevalence of ANA and a low prevalence of anti-SS-A/Ro or SS-B/La antibody were observed in the HAM + SS patients. Although the pathogenesis of SS found in patients with HAM remains unclear, both the preliminary and AECG classification criteria demonstrated similar clinical and serological findings. In our previous study [24], the prevalence of ectopic germinal center (GC) formation along with CXC chemokine ligand-13 (CXCL13) was low in HTLV-I-seropositive patients with SS, especially in HAM patients with SS, indicating that the low prevalence of serology in the present study might be explained by a low prevalence of GCs and CXCL13, because CXCL13 in GCs is an important factor to attract B cells [25, 26] that differentiate into plasma cells and produce autoantibodies such as anti-SS-A/Ro antibody.

Taken together, the past and present findings show that the AECG classification criteria resulted in clinical and pathological features that were similar to those obtained by using the preliminary classification criteria for HTLV-I-seropositive patients with SS. Even though the criteria were revised, our results showed that the HTLV-I-seropositive patients with SS (including HAM patients with SS) had an unwavering tendency with respect to clinicopathological findings. 

## Conclusions

Because we have only fragmentary findings [24, 27] with regard to the pathogenesis of HTLV-I-seropositive patients with SS, a molecular biological approach is necessary to verify the findings obtained in this retrospective study. As a future evaluation, a prospective cohort study including an SS disease activity index such as the European League Against Rheumatism (EULAR) SS disease activity index (ESSDAI) or the EULAR SS patient-reported index (ESSPRI) [28, 29] is required to specifically determine whether HTLV-I is a risk factor of SS and to establish the accurate disease activity of HTLV-I-seropositive patients with SS. As an animal model shows SS-like sialadenitis [20], HTLV-I may be implicated in the pathogenesis of SS. The exclusion of HTLV-I infection from the classification criteria might be considered in the future because human immunodeficiency virus and hepatitis type C infection are also excluded from the AECG and ACR classification criteria.

### Ethics approval

Institutional Review Board of Nagasaki University.

## Abbreviations

HTLV-I: Human T lymphotropic virus type I
HAM: HTLV-I-associated myelopathy
TSP: Tropical spastic paraparesis
ATL: Adult-T cell leukemia
SS: Sjögren’s syndrome
MNC: Mononuclear cell
ACR: American College of Rheumatology
MSGB: Minor salivary gland biopsy
AECG: American European Consensus Group
ELISA: Enzyme-linked immunosorbent assay
FS: Focus score
IQRs: Interquartile ranges
AC: HTLV-I asymptomatic carrier
GC: Germinal center
CXCL13: CXC chemokine ligand-13
SGECs: Salivary gland epithelial cells
TGF-β: Transforming growth factor-beta
EULAR: European League Against Rheumatism

## References

[1] Nakamura T (2009). HTLV-I-associated myelopathy/tropical spastic paraparesis (HAM/TSP): the role of HTLV-I-infected Th1 cells in the pathogenesis, and therapeutic strategy. Folia Neuropathol.

[2] Tsukasaki K, Tobinai K (2013). Biology and treatment of HTLV-1 associated T-cell lymphomas. Best Pract Res Clin Haematol.

[3] Nakamura H, Kawakami A, Eguchi K (2006). Mechanisms of autoantibody production and the relationship between autoantibodies and the clinical manifestations in Sjögren's syndrome. Transl Res.

[4] Fox RI (2005). Sjögren’s syndrome. Lancet.

[5] Miceli-Richard C, Criswell LA (2014). Genetic, genomic and epigenetic studies as tools for elucidating disease pathogenesis in primary Sjögren’s syndrome. Expert Rev Clin Immunol.

[6] Terada K, Katamine S, Eguchi K, Moriuchi R, Kita M, Shimada H (1994). Prevalence of serum and salivary antibodies to HTLV-1 in Sjögren’s syndrome. Lancet.

[7] Tangy F, Ossondo M, Vernant JC, Smadja D, Blétry O, Baglin AC (1999). Human T cell leukemia virus type I expression in salivary glands of infected patients. J Infect Dis.

[8] Sumida T, Yonaha F, Maeda T, Kita Y, Iwamoto I, Koike T (1994). Expression of sequences homologous to HTLV-I tax gene in the labial salivary glands of Japanese patients with Sjögren’s syndrome. Arthritis Rheum.

[9] Shattles WG, Brookes SM, Venables PJ, Clark DA, Maini RN (1992). Expression of antigen reactive with a monoclonal antibody to HTLV-1 P19 in salivary glands in Sjögren’s syndrome. Clin Exp Immunol.

[10] Nakamura H, Eguchi K, Nakamura T, Mizokami A, Shirabe S, Kawakami A (1997). High prevalence of Sjögren’s syndrome in patients with HTLV-I associated myelopathy. Ann Rheum Dis.

[11] Nakamura H, Kawakami A, Tominaga M, Hida A, Yamasaki S, Migita K (2000). Relationship between Sjögren’s syndrome and human T-lymphotropic virus type I infection: follow-up study of 83 patients. J Lab Clin Med.

[12] Vitali C, Bombardieri S, Moutsopoulos HM (1993). Preliminary criteria for the classification of Sjögren’s syndrome: results of a prospective concerted action supported by European Community. Arthritis Rheum.

[13] Vitali C, Bombardieri S, Jonsson R, Moutsopoulos HM, Alexander EL, Carsons SE (2002). Classification criteria for Sjögren’s syndrome: a revised version of the European criteria proposed by the American-European Consensus Group. Ann Rheum Dis..

[14] Shiboski SC, Shiboski CH, Criswell L, Baer A, Challacombe S, Lanfranchi H (2012). American College of Rheumatology classification criteria for Sjögren’s syndrome: a data-driven, expert consensus approach in the Sjögren’s International Collaborative Clinical Alliance cohort. Arthritis Care Res (Hoboken).

[15] Osame M, Usuku K, Izumo S, Ijichi N, Amitani H, Igata A (1986). HTLV-I associated myelopathy, a new clinical entity. Lancet.

[16] Rubin P, Holt JF (1957). Secretory sialography in disease of the major salivary gland. Am J Rentgenol.

[17] Chisholm DN, Mason DK (1968). Labial salivary gland biopsy in Sjögren’s disease. J Clin Pathol.

[18] Greenspan JS, Daniels TE, Talal N, Sylvester RA (1974). The histopathology of Sjögren’s syndrome in labial salivary gland biopsies. Oral Surg Oral Med Oral Pathol.

[19] Nakamura H, Kawakami A, Izumi M, Nakashima T, Takagi Y, Ida H (2005). Detection of the soluble form of Fas ligand (sFasL) and sFas in the saliva from patients with Sjogren’s syndrome. Clin Exp Rheumatol..

[20] Green JE, Hinrichs SH, Vogel J, Jay G (1989). Exocrinopathy resembling Sjögren’s syndrome in HTLV-1 tax transgenic mice. Nature.

[21] Hida A, Imaizumi M, Sera N, Akahoshi M, Soda M, Maeda R (2010). Association of human T lymphotropic virus type I with Sjogren syndrome. Ann Rheum Dis.

[22] Iwanaga M, Watanabe T, Utsunomiya A, Okayama A, Uchimaru K, Koh KR (2010). Human T-cell leukemia virus type I (HTLV-1) proviral load and disease progression in asymptomatic HTLV-1 carriers: a nationwide prospective study in Japan. Blood.

[23] Hisada M, Okayama A, Shioiri S, Spiegelman DL, Stuver SO, Mueller NE (1998). Risk factors for adult T-cell leukemia among carriers of human T-lymphotropic virus type I. Blood.

[24] Nakamura H, Kawakami A, Hayashi T, Nakamura T, Iwamoto N, Yamasaki S (2009). Low prevalence of ectopic germinal centre formation in patients with HTLV-I-associated Sjogren’s syndrome. Rheumatology (Oxford).

[25] Ansel KM, Ngo VN, Hyman PL, Luther SA, Förster R, Sedgwick JD (2000). A chemokine-driven positive feedback loop organizes lymphoid follicles. Nature.

[26] Shi K, Hayashida K, Kaneko M, Hashimoto J, Tomita T, Lipsky PE (2001). Lymphoid chemokine B cell-attracting chemokine-1 (CXCL13) is expressed in germinal center of ectopic lymphoid follicles within the synovium of chronic arthritis patients. J Immunol.

[27] Nakamura H, Takahashi Y, Yamamoto-Fukuda T, Horai Y, Nakashima Y, Arima K (2015). Direct infection of primary salivary gland epithelial cells by human T lymphptropic virus type I in patients with Sjögren’s syndrome. Arthritis Rheumatol.

[28] Seror R, Mariette X, Bowman S, Baron G, Gottenberg JE, Bootsma H (2010). Accurate detection of changes in disease activity in primary Sjögren’s syndrome by the European League Against Rheumatism Sjögren’s Syndrome Disease Activity Index. Arthritis Care Res (Hoboken).

[29] Seror R, Bootsma H, Saraux A, Bowman SJ, Theander E, Brun JG et al. Defining disease activity states and clinically meaningful improvement in primary Sjögren’s syndrome with EULAR primary Sjögren’s syndrome disease activity (ESSDAI) and patient-reported indexes (ESSPRI). Ann Rheum Dis. 2014: Dec 5. [Epub ahead of print]. 10.1136/annrheumdis-2014-206008 25480887

